# *In Situ* Field Sequencing and Life Detection in Remote (79°26′N) Canadian High Arctic Permafrost Ice Wedge Microbial Communities

**DOI:** 10.3389/fmicb.2017.02594

**Published:** 2017-12-20

**Authors:** J. Goordial, Ianina Altshuler, Katherine Hindson, Kelly Chan-Yam, Evangelos Marcolefas, Lyle G. Whyte

**Affiliations:** ^1^Department of Natural Resource Sciences, McGill University, Ste. Anne-de-Bellevue, QC, Canada; ^2^Bigelow Laboratory for Ocean Sciences, East Boothbay, ME, United States

**Keywords:** life detection, astrobiology, nanopore MinION, polar microbiology, permafrost

## Abstract

Significant progress is being made in the development of the next generation of low cost life detection instrumentation with much smaller size, mass and energy requirements. Here, we describe *in situ* life detection and sequencing in the field in soils over laying ice wedges in polygonal permafrost terrain on Axel Heiberg Island, located in the Canadian high Arctic (79°26′N), an analog to the polygonal permafrost terrain observed on Mars. The life detection methods used here include (1) the cryo-iPlate for culturing microorganisms using diffusion of in situ nutrients into semi-solid media (2) a Microbial Activity Microassay (MAM) plate (BIOLOG Ecoplate) for detecting viable extant microorganisms through a colourimetric assay, and (3) the Oxford Nanopore MinION for nucleic acid detection and sequencing of environmental samples and the products of MAM plate and cryo-iPlate. We obtained 39 microbial isolates using the cryo-iPlate, which included several putatively novel strains based on the 16S rRNA gene, including a *Pedobacter* sp. (96% closest similarity in GenBank) which we partially genome sequenced using the MinION. The MAM plate successfully identified an active community capable of L-serine metabolism, which was used for metagenomic sequencing with the MinION to identify the active and enriched community. A metagenome on environmental ice wedge soil samples was completed, with base calling and uplink/downlink carried out via satellite internet. Validation of MinION sequencing using the Illumina MiSeq platform was consistent with the results obtained with the MinION. The instrumentation and technology utilized here is pre-existing, low cost, low mass, low volume, and offers the prospect of equipping micro-rovers and micro-penetrators with aggressive astrobiological capabilities. Since potentially habitable astrobiology targets have been identified (RSLs on Mars, near subsurface water ice on Mars, the plumes and oceans of Europa and Enceladus), future astrobiology missions will certainly target these areas and there is a need for direct life detection instrumentation.

## Introduction

The search for life on other solar system bodies is and will be a major focus of planetary exploration in the coming decades. The primary targets for astrobiology investigations of other solar system bodies are Mars, in the short term, as well as Europa and Enceladus, in the mid to longer term (Hays et al., 2015). Extremely cold temperatures characterize these primary astrobiology targets, and, as such, the best terrestrial analogs may be the Earth's polar regions. Based on our current knowledge of extremophile microbiology, these targets are potentially capable of hosting microbial life and ecosystems either currently or in the past. For example, considerable evidence has been found on Mars indicating that, before ~3.5 bya, the planet was warmer and wetter (Golombek, 1999). The Mars Science Laboratory (MSL) mission has reported ample evidence of past fluvial, deltaic, and lacustrine environments within Gale Crater (Grotzinger et al., 2014); However, the recent report of surface brine water at several reoccurring slope linaea (RSL) locations on Mars (Ojha et al., 2015) now opens up the possibility that extant microbial life (which would be most likely cold-adapted and halophilic) could be present at these sites and will almost certainly be the targets of future missions in the mid-2020s and beyond, including potential sample return missions. Similarly, exciting discoveries over the last 5–10 years point to the existence of cold, salty oceans under the ice covers of Europa and Enceladus (Kargel et al., 2000; Melosh et al., 2004; Waite et al., 2006; McKay et al., 2008; Roth et al., 2014) which could also support extant microbial ecosystems based on our knowledge of similar salty cryoenvironments on Earth (Goordial et al., 2013).

Currently, the scientific instruments available for astrobiology space missions are focused on identification of habitable environments or on detection of biosignatures. To date, these instruments remain high mass, large in size, and have high energy requirements. Such instruments are entirely unsuited for missions to locations such as Europa or Enceladus for which lander packages are likely to be tightly constrained. Even for Mars missions, there are advantages to utilizing multiple small scientific instruments over fewer larger instruments. Additionally, the difficulties in defining biomarkers attest to the need for more specific astrobiological instruments to be deployed (Brasier et al., 2004). Though life detection is a primary driver of planetary exploration, no direct life detection instrumentation has been included on an astrobiology mission payload since the Viking missions to Mars in the 1970's (Levin, 1997; Davila et al., 2010; Schulze-Makuch et al., 2015).

In the present study, we utilized pre-existing, low mass, low cost, and robust next generation miniaturized scientific instrumentation for biosignature detection, and microbiology techniques in a novel context for biosignature identification and characterization of viable microbial life in a high fidelity analog environment in the Canadian high Arctic. Such a MICRObial life detection platform attached to surface rover platforms and/or penetrators could conceptually be used in future missions to Mars, Europa, Enceladus or any other planetary target. Additionally, such a platform also has clear applications for quick analysis of community metagenomics and assessment of microbial activity in environmental field sites, including extreme sites such as the high Arctic and Antarctic (Edwards et al., 2016; Johnson et al., 2017).

**Figure 1:**
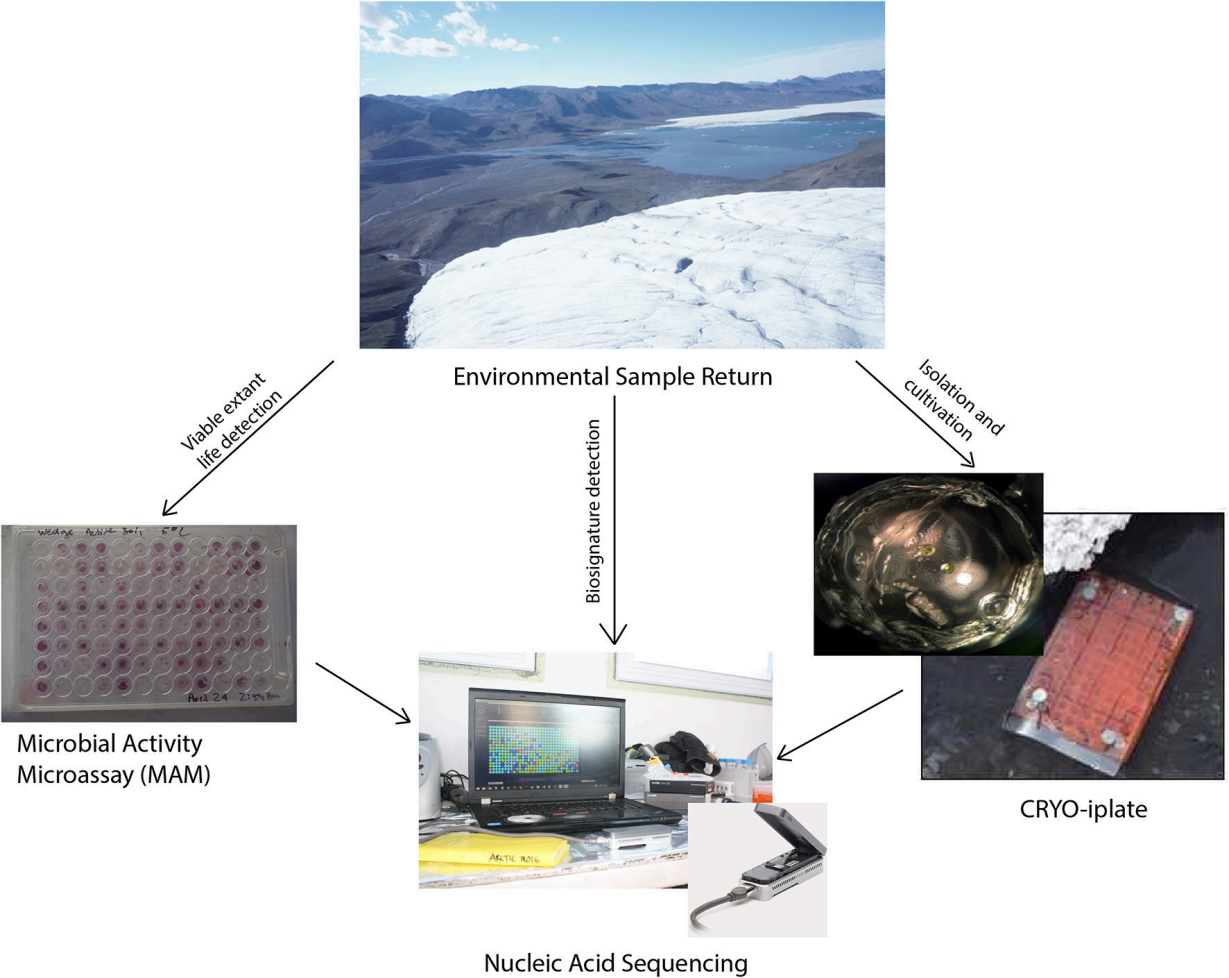
MICRO life detection platform components tested.

The conceptual life detection platform we developed and tested here is modular, and it is envisioned that as new technologies and methodologies emerge that they can be “swapped out” or added to such a platform. The life detection platform components tested here includes: (Hays et al., 2015) the cryo-iPlate for culturing microorganisms using diffusion of *in situ* nutrients on a solid media based on the recently described innovative ichip method (Golombek, 1999; Nichols et al., 2010) a microbial activity microassay (MAM) (Ecolog plate) for detection of viable microoganisms through a colourimetric assay, and (Grotzinger et al., 2014) the Oxford Nanopore MinION for biosignature detection (DNA, RNA, and in the near future proteins) which can be used for environmental samples, as well as for positive results from the cryo-iPlate and positive wells in the microbial activity assay (Figure 1).

## Materials and methods

### Study site and sampling

The analog study site is located near the McGill Arctic Research Station (MARS) on Axel Heiberg Island in the Canadian High Arctic (79°26′N, 90°46′W). MARS is located in a polar desert and is located in proximity to a number of high fidelity Mars analog sites of interest to astrobiology, including cold saline springs arising through permafrost, gypsum cryptoendoliths and extensive polygonal permafrost terrain underlain with ice wedges similar in structure to those observed on Mars (Pollard et al., 2009; Fairén et al., 2010). Soils are a low carbon mineral cryosol, with a mean annual temperature of −15.2°C (Doran, 1993; Lau et al., 2015). The active soil layer that overlays the permafrost ranges 60-73 cm in depth during the summer and is completely frozen over during winter and spring months. For this study, soil samples overlaying the ice-wedges were aseptically collected using an ethanol-sterilized metal sampling spoon to scrape the frozen trough soils into sterile 50 mL falcon tubes.

### DNA extraction

*Ice-wedge soil:* DNA was extracted from two 0.5 cc of thawed ice wedge soil using the PowerLyzer (MoBio Laboratories, Inc., Carlsbad, CA) according to manufacturer's protocol. The two replicates were pooled prior to sequencing. This required the use of a vortex and centrifuge in the field, in addition to pipettors and consumables. *Biolog plate: Three* replicate wells which showed microbial metabolism on L-serine were chosen for extraction. The three wells were pooled (total 600 μl), and added directly to a bead tube from the MoBio PowerLyzer kit, and processed according to manufacturer's instruction. The DNA extractions for the Biolog plate and Cryo-iplate were quantified in the field using a Qubit 2.0 Fluorometer (Life Technologies) per the manufacturer's instruction, and measured to be 13.3 and 4.6 ng/μL (total volume extracted 90 ul). *Cryo-iplate Pedobacter isolate*: The *Pedobacter* sp. was isolated as described below. In the lab, the strain was grown in R2A broth for 1 week, and the culture pelleted and resuspended in 500 μL water for use with the PowerLyzer kit. DNA extracted was 307 ng/μL (90 μL volume) measured on a NanoDrop instrument (Thermo Scientific, Wilmington, MA).

### MinION sequencing and analysis

All reagents were stored in a cooler with blue ice for transportation from McGill University (Montreal, Canada) to the McGill Arctic Research Station. *DNA Library Preparation:* At MARS we used the MinION MK1 device for sequencing with the Nanopore Sequencing Kit (SQK- MAP006) combined with the with EXP-LWI001(Low Input Expansion Kit). These kits were used according to manufacturer's instruction, but with the omission of the DNA shearing step. Briefly, 46 μL of DNA extract was used for a DNA repair step using NEBNext FFPE Repair Mix (New England Biolabs, Ipswich, MA), followed by clean-up using AMPure XP beads incubated with mixing at room temperature for 5 min. Samples were ethanol cleaned while on a magnetic rack. Water was then added to beads, and the 45 μL eluate used for end repair and dA-tailing of DNA using NEBNext Ultra II End-repair/dA tailing kit (New England Biolabs). The end repaired DNA was washed again on a magnetic rack with ethanol, and eluted with 10 μL nuclease free water. The 10 μL of eluate was used for adapter ligation using the NEB Blunt/TA Ligase Master Mix (New England Bioloabs) with the Low Input Hairpin Tether from the low input expansion pack (EXP-LWI001). The adapted and tethered DNA library was subsequently bound to purified MyOne C1 Streptavidin beads and suspended in resuspension buffer provided with the library prep kit. The beads were loaded onto MinION Flow Cells (R7.3) according to protocol, and sequenced for 12 h powered by a laptop, with intermittent battery charging power supplied through a generator. In the lab, we used the MinION rapid library prep kit (SQK-RAD001), in conjunction with the MinION MK1b device for library preparation according to manufacturer's instruction, and using MinION Flow Cells (R9), again omitting the DNA shearing step. Samples were sequenced over 48 h. *Sequence Analysis:* Base-calling was performed using Metrichor software (Oxford Nanopore). Fasta files were extracted from the generated fast5 files using PoreTools (Loman and Quinlan, 2014). Passed 2D and 1D reads from the ice-wedge soil sample and Biolog plate were uploaded to MG-RAST (Meyer et al., 2008), and sequences additionally quality trimmed using the default settings for dynamic trimming (sequences contain <5 bp below a phred score of 15). Any human or chordata contaminants were removed from the dataset. To determine taxonomy/functional genes, we used RefSeq annotated proteins in MG-RAST (*e*-value ≤ 10^−5^, 60% protein similarity, alignment length >15). We removed reads identified as belonging to *E. Coli* from further analysis (6,131, 55, 3 genes identified in the rapid kit ice-wedge metagenome, low input kit ice-wedge metagenome, and Biolog plate metagenome respectively). Metagenome sequences are deposited in MG-RAST under accession numbers mgm4705800.3, mgm4705797.3, mgm4735353.3, and mgm4718581.3. The *Pedobacter* isolate genome sequence was base called and the fastq sequences extracted as for the metagenomes. 9050 reads were assembled into 87 contigs using the long read assembler Canu (release 1.4) (Koren et al., 2017) using an assumed genome size of 5 Mb, based on the average genome sizes of closely related *Pedobacter* sp. genomes on the JGI Integrated microbial genomes (IMG) database. Assembled reads have been deposited at DDBJ/ENA/GenBank under the accession PEIM00000000. The version described in this paper is version PEIM01000000. Assembled contigs were annotated using MyRast (Overbeek et al., 2013), and those annotations scanned for genes associated with cold adaptation.

### Illumina sequencing

Community bacterial 16S rRNA amplicon sequencing was performed using an Illumina MiSeq (Illumina, San Diego, CA, USA) following the 16S rRNA gene sample preparation protocol. Amplicon primers targeted the V3 and V4 regions with the Illumina adapter overhand sequences (forward: 5′-TCGTCGGCAGCGTCAGATGTGTATAAGAGACAGCCTACGGGNGGCWGCAG-3′; reverse: 5′-GTCTCGTGGGCTCGGAGATGTGTATAAGAGACAGGACTACHVGGGTATCTAATCC-3′) from Integrated DNA Technologies (Coralville, IA, USA). For the amplicon PCR, each 25 μL reaction contained 2.5 μL sample, 8.5 μL PCR-grade water, 12.5 μL 2× KAPA HiFi HotStart ReadyMix (KAPA, Wilmington, MA, USA), and 0.75 μL of 20 μM forward and reverse primers. PCR was performed with a 95°C initialization for 5 min, followed by 30 cycles of 95°C for 30 s, 55°C for 30 s, 72°C for 45 s, and a final 72°C extension for 10 min. The results were then verified on a gel. For the Index PCR step, index primers from the Nextera XT Index Kit for 24 samples (Illumina, San Diego, CA, USA) were used. The 25 μL sequencing reaction contained 12.5 μL2× KAPA HiFi HotStart ReadyMix, 2.5 μL PCR-grade water, 2.5 μL of each primer 1 and 2, and 5 μL purified amplicon. Indexing PCR was performed with a 95°C initialisation for 5 min, followed by 8 cycles of 95°C for 30 s, 55°C for 30 s, 72°C for 30 s, and a final 72°C extension for 5 min. After clean-up, the PCR products were quantified by Qubit fluorometric quantification with the Qubit dsDNA BR Assay Kit (Thermo Fisher Scientific, Waltham, MA, USA). The indexed library was pooled to about 4 nM and the quality was checked with an Agilent DNA 1000 kit and Agilent 2100 Bioanalyzer (Agilent Technologies, Santa Clara, CA, USA). The pooled library was run on the Illumina MiSeq platform with the MiSeq Reagent Kit v3, 600 cycles. The pooled denatured library was diluted to 4 pM as recommended by the protocol.

### Isolation of permafrost bacteria using a prototype cryo-iplate methodology

The design of the cryo-iplate used in this study was largely inspired by Nichols et al.'s isolation chip (ichip) methodology (Nichols et al., 2010). Semi-permeable hydrophilic polycarbonate membranes with 0.03 μm pores (Whatman plc GE Healthcare Life Sciences, Mississauga, ON, Canada) were glued with silicon to the bottom of 96 well trays, obtained by disassembling a 200 μl-pipette-tip boxes (FroggaBio, North York, ON, Canada)—providing a barrier between the plate wells and the external environment. The assembly was then autoclaved to sterilize. The 96 wells of the sterilized tray were then filled with 2% w/v gellan gum (Alfa Aesar, Haverhill, Massachusetts, USA) in the laboratory prior to field deployment. In the field, the top of each gellan-filled well of the central tray was inoculated with an estimated ~1 microbial cell using 10 μL of a 10^−8^ diluted ice wedge soil solution based on our previous study of this polygon site (Wilhelm et al., 2011, 2012). Once inoculated, the cryo-iplate assembly was sealed with a sterile 96 well plate film. One cryo-iplate was incubated *in situ* at the study site in the wedge soil for 14 days in May 2016. A second cryo-iplate was incubated at room temperature in a whirlpak bag filled with thawed ice-wedge soil. Both cryo-iplates were transported back to McGill in a cooler and held at 5°C, until processing within 1 month. In the laboratory, gellan plugs were removed from trays of both cryo-iplates using sterile toothpicks and were then observed under a dissecting microscope for microcolony formation. Observable colonies were picked using a toothpick and streaked onto ½ R2A broth media (Difco, USA) supplemented with 2% w/v gellan and incubated for 14 days at 10°C. Successive rounds of subcultivation at 20°C on solid ½ R2A media were conducted to isolate distinct morphotypes.

#### Isolate identification

For colonies isolated from the Cryo-iPlate that were subcultivatable, cell lysates were prepared by placing biomass from agar plates in 250 μL of molecular grade dH_2_0 (ThermoFisher Scientific). Lysis was achieved through vortexing and heating in a microwave oven for 3 min before centrifugation for 30 s at 10.0 RCF. Each PCR reaction consisted of a final volume of 20 μL containing: 200 μM dNTP, 0.5 μM of each primer (27F/1492R for bacterial lysates and ITS1F/R for 18S amplification of fungal lysates), 2.5 U/reaction of *HotStarTaq Plus* DNA Polymerase (Qiagen), ~1 μg template DNA, and 1.5 mM MgCl_2_. The final concentrations above followed Qiagen's HotStartTaq Kit instructions. The sequence of the 27F (forward) bacterial primer consisted of: 5′-AGAGTTACCTTGTTACGACTT-3′. The sequence of the 1492R (reverse) bacterial primer was: 5′-GGTTACCTTGTTACGACTT-3′. The 16S rDNA amplification PCR reaction cycling program consisted of: (1) 95°C for 7 min, (2) 94°C for 45 s, (3) 55°C for 45 s, (4) 72°C for 1 min, (where steps 2–4 were repeated 30 times), (5) 72°C for 10 min. Amplification of the 16S rRNA gene was confirmed on an agarose gel. Sanger sequencing using the 27F primer was conducted at the Plate-forme d'Analyses Génomiques sequencing center at Laval University. For quality control, chromatograms were first manually curated using 4Peaks software (http://nucleobytes.com/4peaks/) where sequence segments with an average Q>40 was used as a query against the NCBI non-redundant database (https://blast.ncbi.nlm.nih.gov/). The 16S rRNA gene sequences are publicly available on GenBank with accession numbers MG266397–MG266429.

### Biolog characterization of microbial communities

We used Eco-plates (Biolog, Hayward, CA, USA) for colourimetric evaluation of community metabolic activity. Wedge soil was added to a 50 mL falcon tube up to a 5 mL volume. The tube was filled to 45 mL with sterile water. The tube was vortexed for 30 s and particles allowed to settle for 10 min. The supernatant was used to inoculate wells of the Ecoplate (150 μL per well), as well as to inoculate iplates. Eco-plates were incubated at 20° and 5°C for 10 days, and color formation monitored daily based on visualization and photographed with a Sony ILCE-6000. After 3 days of incubation, L-serine wells from the 20°C incubation were pooled, cells pelleted at 10,000 × g for 5 min, and resuspended in 500 μL of water. The re-suspended cells were then used for a DNA extraction as described above.

## Results

### Nucleic acid life detection in ice-wedge soils

**Figure 2:**
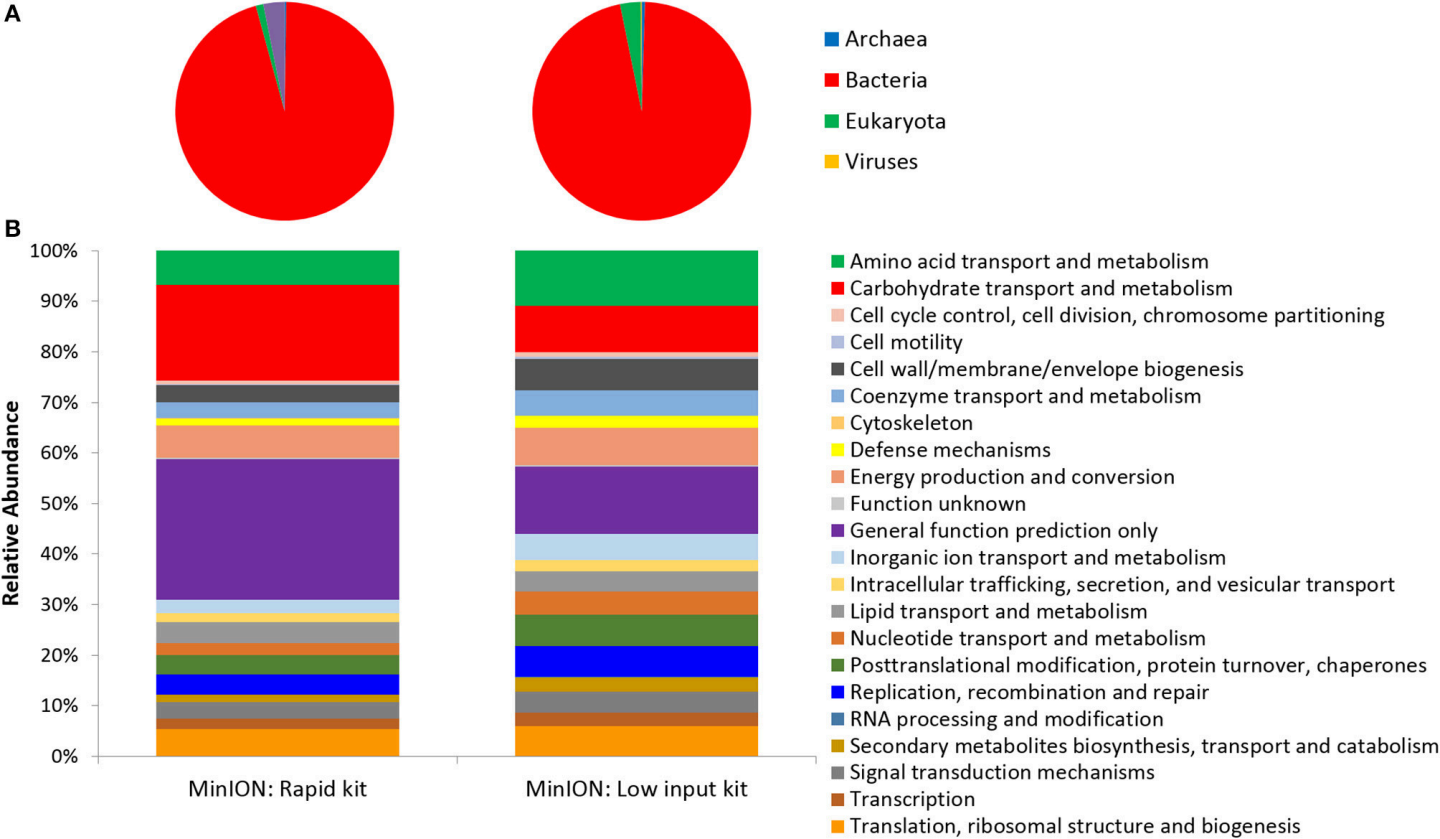
Function and Taxonomy of Ice Wedge soil. Ice wedge soil metagenomes sequenced using the MinION rapid and low input kit **(A)** Taxonomy at the domain level **(B)** Clusters of Orthologous Groups (COG) categories (level 2).

**Figure 3:**
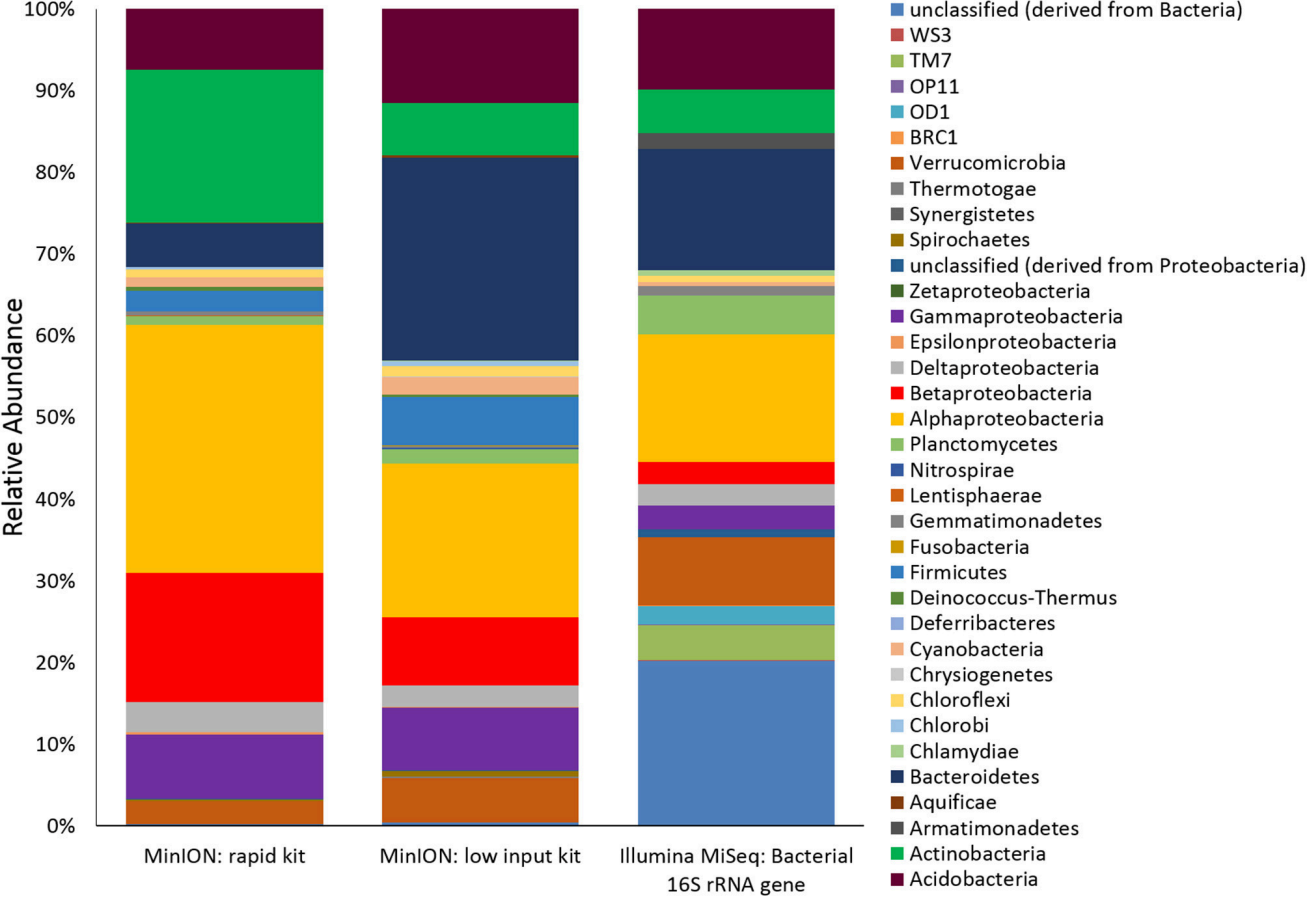
Ice Wedge soil bacterial community composition detected by the MinION. Bacterial reads from ice wedge soil metagenomes sequenced using the MinION rapid and low input kit compared with Bacterial composition inferred from amplicon sequencing of 16S rRNA gene.

Two metagenomes were sequenced from a single ice-wedge soil DNA extract using the MinION rapid library prep kit (SQK-RAD001) and the MinION low input library prep kit (SQK-NSK007 combined with the Low Input Expansion Kit; EXP-LWI001). The MinION rapid library preparation kit requires fewer consumables, and took ~20 min to complete, but requires ~200–1,000 ng/μL of DNA. The MinION low input library preparation kit required much lower amounts of DNA (~20–100 ng), took ~160 min and required more instrumentation (a microcentrifuge, magnetic beads, additional enzymes, ligation of a hairpin adapter etc.) Both methods generated single molecule reads without the need for PCR amplification and resulted in similar functional and taxonomic profiles of the ice-wedge soil community (Figure 2). Sequencing results are summarized in Table S1, the low input kit generated 6,348 sequences with a mean length of 3,811 ± 2,704, and the rapid kit generated 9,530 sequences of 3,018 ± 2,015 bp in length. The ice wedge soil was dominated by bacteria (~96%) using both library preparation kits, with low, but detectable sequences belonging to Archaea (0.3–05% of reads). The MinION rapid kit detected more reads annotated as viral in origin (3% of reads), and less Eukaryotic sequences (1%) than the MinION low input kit on the same DNA extract (0.2 and 3% respectively). The McGill Arctic research station (MARS) is equipped with satellite internet, that works intermittently and is highly weather dependent. Due to the stochastic nature of internet access and poor weather during the latter half of the expedition at MARS we were only able to carry out one data transfer of a MinION sequencing run in which raw data was uplinked to the cloud-based base calling service, Metrichor (Oxford Nanopore), and the base called nucleic acid sequences downlinked back to MARS. A local base calling program that was provided by Oxford Nanopore upon request was used in the Arctic for the other sequencing runs carried out at MARS. To evaluate the accuracy of the MinION sequencing data obtained in the field, we extracted only Bacterial reads from the MinION metagenomic data sets and compared the assigned taxa with Bacterial 16S rRNA gene amplicon sequences (Illumina MiSEQ) generated from the same DNA extract used in the field with the MinION. At the phyla level, similar taxonomic groups were identified from Illumina sequencing and with the MinION (Figure 3), however the relative abundances varied between the two library preparation kits, as well as the amplicon sequencing. There was a larger number of unclassified reads associated with the amplicon dataset, possibly attributable to the longer read lengths derived from the MinION. *Alphaproteobacteria, Actinobacteria, Acidobacteria*, and *Bacteriodetes* were the prominent phyla present in all three datasets, consistent with previous molecular surveys in permafrost ice-wedge soils at this site (Wilhelm et al., 2011). The metagenomes derived from the MinION identified proteins clustered into 22 COG categories, which similarly varied in relative abundance between library preparation kits (Figure 2). We used the MinION generated ice-wedge soil metagenomes to identify functional genes and pathways in the microbial communities. For example, we were able to observe all of the genes in the (ubiquitous) citric acid cycle (TCA) (Figure S1). We also identified genes known to be associated with cold adaptation in permafrost microorganisms (Goordial et al., 2016b, 2017) (ie- genes for osmotic stress response mediated through compatible solutes like glycine betaine and choline, cold shock proteins, oxidative stress) (Table S2).

### Viable microbial activity assay using the biolog ecoplate

**Figure 4:**
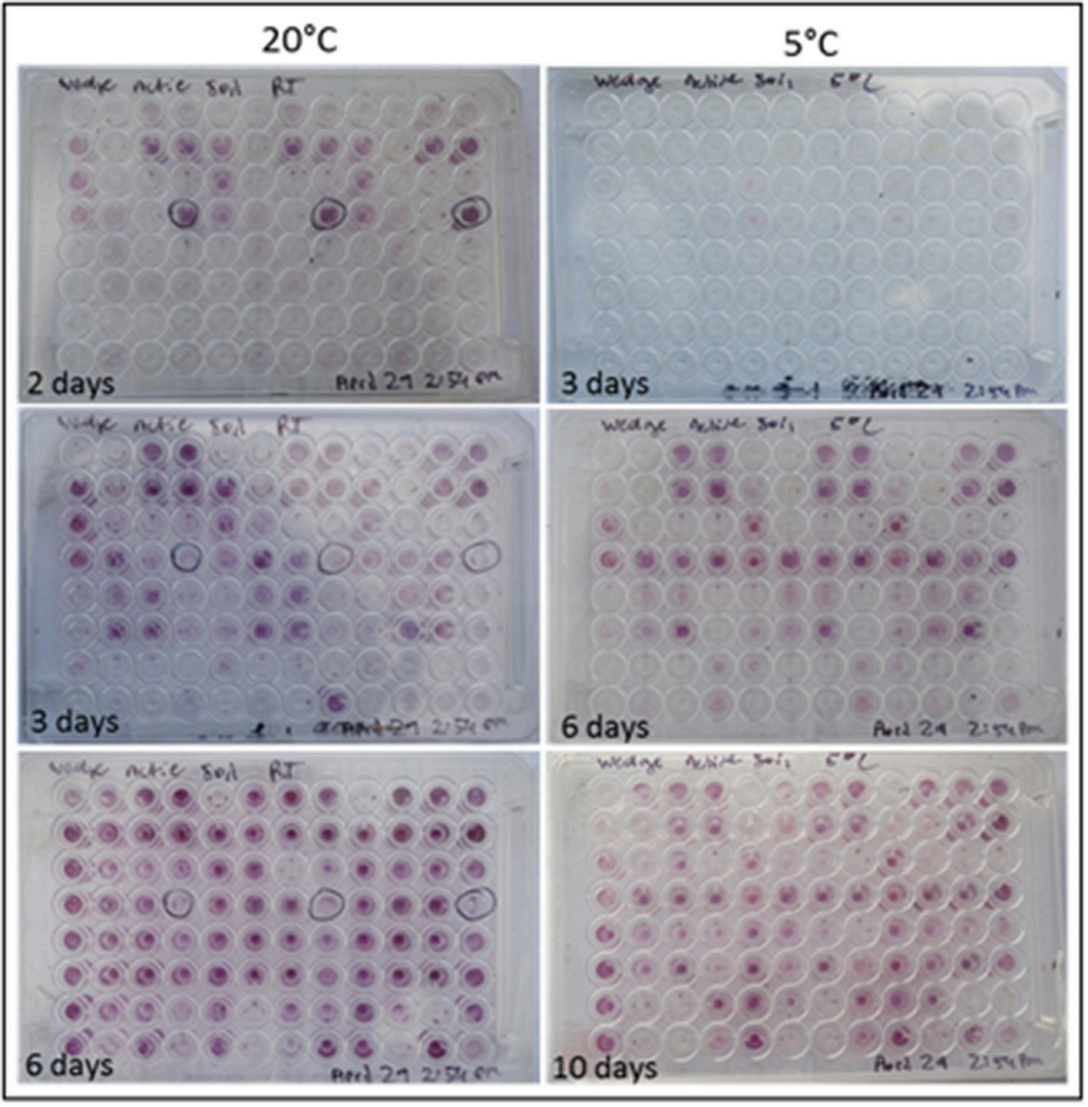
Colorimetric microbial metabolic activity assay (Ecolog). Ecolog plates over several days incubation at 20° and 5°C. Colored wells in Ecolog plates are indicative of microbial activity.

**Figure 5:**
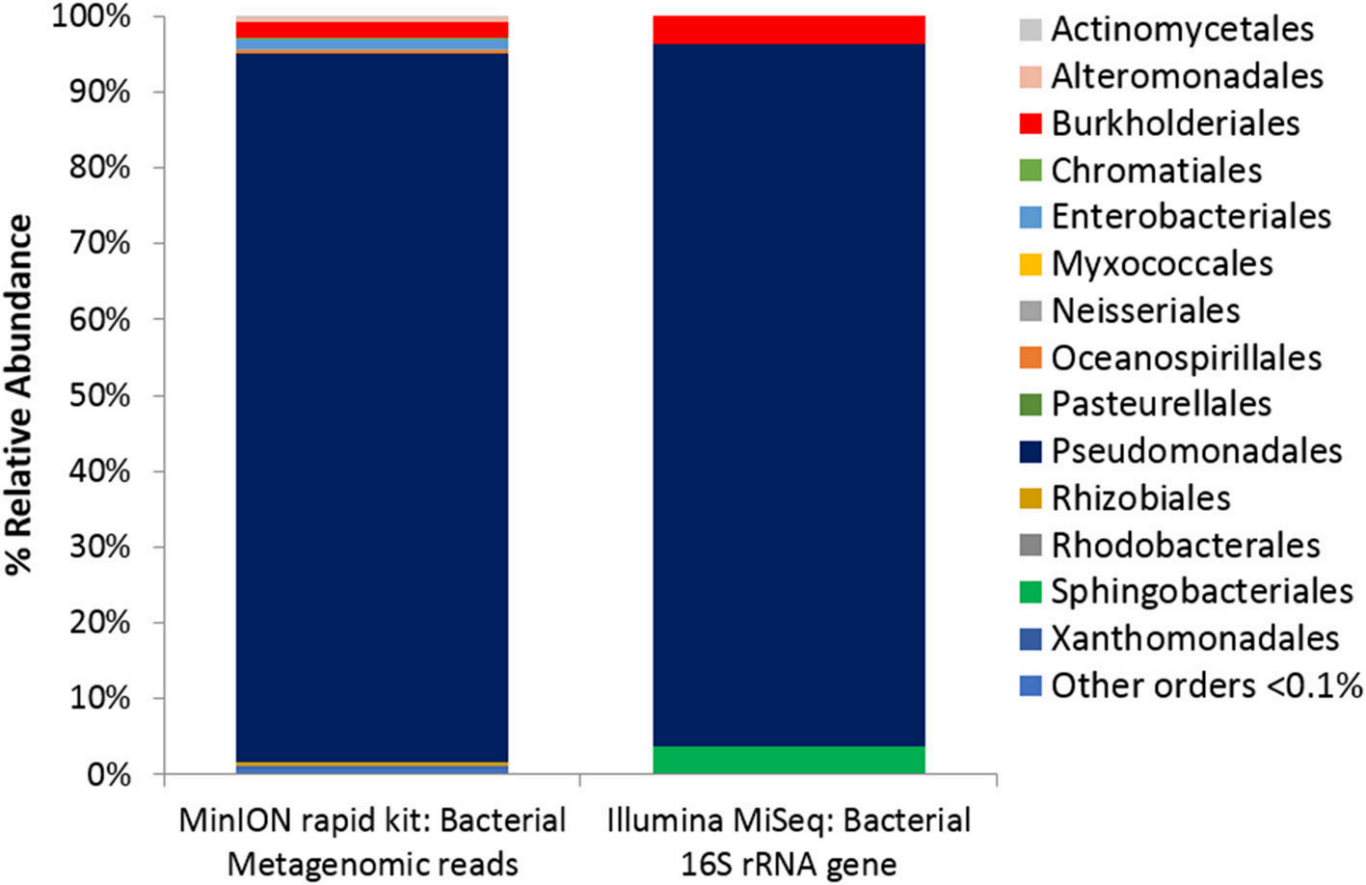
Microbial Activity Microassay (Ecolog plate) Bacterial diversity. Bacterial reads from Ecolog metagenome sequenced using the MinION rapid kit compared with Bacterial composition inferred from amplicon sequencing of 16S rRNA gene.

Colorimetric assays such as those employed in the Biolog Ecoplate enable easy and fast visualization of microbial growth based on a colorimetric reaction when a carbon substrate is reduced for growth. Such assays can be miniaturized, and made to be high throughput, with community level physiological profile (CLPP) interpreted on a basic level via visualization alone and quantified via a spectrometer (Weber and Legge, 2009; Rutgers et al., 2016). Slurries of ice wedge soil and sterilized water were used as inocula for the Biolog Ecoplate, CLPP were obtained at 20° and 5°C. We attempted to incubate Ecoplates in the field however all wells froze. While not tested here, the use of freezing point depressants such as NaCl or glycerol in the wells could enable identification and enrichment of active, cold-tolerant and osmo-tolerant microorganisms *in situ*. Wells were assessed visually alone as we did not have access to a spectrophotometer in the field. Substrate utilization patterns were faster at 20°C for all substrates that tested positive (Figure 4), color formation was visible in the 20°C plate after 24 h incubation, and only visible in the 5°C plate after 3 days. There were no substrates which could be utilized by a cold adapted community incubated at 5°C that did could not be utilized by a community capable of metabolism at 20°C (Table 1), the order of substrate utilization over time was not the same for both communities. For example, L-serine, L-arginine, 2-hydroxy benzoic acid, 4-hydroxy benzoic acid were among the first substrates to be visibly reduced in the 5°C incubation, occurring nearly simultaneously (4 days); In the 20°C incubation L-serine was visibly used almost immediately (8 h), while L-arginine, 2-hydroxy benzoic acid, 4-hydroxy benzoic acid proceeded relatively slowly after that (3 days). These differences are likely attributable to different metabolically active microbial communities with different temperature tolerances and optima in the ice wedge soil. We selected reduced wells containing the amino acid L-serine due to the speed and intensity of color formation in these wells after 2 days at 20°C. The wells were pooled together, and DNA extracted using a conventional DNA extraction kit (MoBIO power soil) before sequencing with the MinION low input kit. These wells generated no “passed” sequences which were above the default quality cut-offs generated by the Nanopore Metrichor software after a 6 h sequencing run. Post-deployment, the same DNA extract was sequenced successfully using the MinION rapid library prep. The reason for this is not clear, it is possible that inhibitors associated with the redox dye chemistry disrupted sequencing with the low input kit due to the use of direct binding of beads to the nanopores, compared with the rapid kit which does not employ beads. The L-serine wells were composed almost entirely (~90%) of a *Pseudomonas*, results which were confirmed via Illumina sequencing of the 16S rRNA gene (Figure 5). The MinION generated metagenome of the L-serine ecolog well included full ORFs which encoded for proteins related to conversion of serine to amino acids cysteine, glycine, and methionine. We identified a serine transporter on a single molecule read (ORF 90% similar to an ORFs found in *Pseudomonas antarctica* PAMC27494; Genbank accension CP015600.1) and searched the GenBank database against the entire read. This single molecule read was 5005 bp in length with 89% similarity to the *P. antarctica* PAMC 27494 genome across 95% of the query; only the last 202 bp of the read did not match to the *P antarctica* genome. This included homology to 3 hypothetical proteins in *P. Antarctica* PAMC27494, 5 intergenic regions, and a partial serine dehydratase ORF (Figure S2). Another single molecule read with serine metabolism related hits (3,606 bp) had 87% similarity to the *P. Antarctica* genome (97% query cover), this single molecule read alignment included homology to 3 intergenic regions.

**Table 1:** Carbon substrate usage in ice wedge soils (6 day incubation).

**Carbon substrate**	**20°C**	**5°C**
2-Hydroxy Benzoic Acid	+	−
4-Hydroxy Benzoic Acid	++	++
D-Galacturonic Acid	+++	++
D-Glucosaminic Acid	+++	+
D,L-α-Glycerol Phosphate	+	−
D-Cellobiose	+++	−
D-Galactonic Acid γ-Lactone	+++	++
D-Malic Acid	+++	−
D-Mannitol	++	++
D-Xylose	+++	−
Glucose-1-Phosphate	++	−
Glycogen	+++	−
Glycyl-L-Glutamic Acid	+++	−
i-Erythritol	+	−
Itaconic Acid	+++	++
L-Phenylalanine	+	−
L-Arginine	+++	++
L-Asparagine	+++	++
L-Serine	+++	++
L-Threonine	++	−
N-Acetyl-D-glucosamine	++	+
Phenylethylamine	+	++
Putrescine	+	+
Pyruvic Acid Methyl Ester	+++	−
Tween 40	+++	++
Tween 80	+++	++
Water	+	−
α-Cyclodextrin	+++	−
α-D-Lactose	+++	−
α-Ketobutyric Acid	++	−
β-Methyl-D-glucoside	+++	−
γ-Hydroxybutyric Acid	++	+

### Cryo-iPlate culturing and analyses

From the cryo-iPlates 33 bacterial isolates were successfully sub-cultured and chosen for sequencing based on differences in morphology and color (Table 2). Most of these isolates were related to strains previously cultured, and 3 isolates were candidates for novel species based on 16S rRNA gene sequence (<98% similarity to anything in GenBank database). It is quite probable that a higher number of slow-growing novel microorganisms would be isolated if longer incubation times for the cryo-iPlates were included in this study (greater than 2 weeks) based on in-laboratory incubations of cryo-iPlates with environmental samples from other Arctic environments (ex- saline sprig sediments) (data not shown). Based on the initial 16S rRNA gene sequencing results, one of the isolates, a *Pedobacter* sp. Str. IW39 (closest cultured representative similarity 96%) was sequenced using the MinION on a single flow cell over 48 h. Genome sequencing was carried out in the laboratory post-field deployment, while the initial colony on a cryo-iplate was in hand in the field. A partial genome was obtained, consisting of 9050 reads, and the majority of reads were <2,000 bp in length, and the longest read was 31,623 bp in length (Figure S3). The longest read was found to be most similar (77%) to *Pedobacter cryoconitis* PAMC 27485, a soil isolate from King George Island, Antarctica (GenBank: CP014504.1). Using Canu, a long read assembler (Koren et al., 2017), we assembled the *Pedobacter* genome into 87 contigs. We were able to identify several genes associated with cold adaptation/osmotic stress regulation such as two glycerol uptake facilitator proteins, a glycerol kinase, and glycerol 3-phosphate dehydrogenase occurring in close proximity to each other. Glycerol is a known compatible solute that depresses freezing point, protects cells from damage caused by freezing, and is synthesized in cultures grown at subzero temperatures (Bore et al., 2017).

**Table 2:** Cryo-iplate subcultured isolates.

**Strain ID #**	**GenBank accession no**.	**Closest cultured BLAST match**	**% Identity**	**Isolation source [Accession no.]**	**Closest BLAST match**	**% Identity**	**Isolation source**
IW5	MG266397	Flavobacterium sp. UA-JF1530	96	glacial river Iceland: Jokulsa a Fjollum [KC108930.1]	Flavobacterium sp. UA-JF1530	96	glacial river Iceland: Jokulsa a Fjollum [KC108930.1]
IW39	MG266398	Pedobacter sp. UYP1	96	Endolythic, Antarctica: King George Island [KU060818.1]	Pedobacter sp. UYP1	96	Endolith, Antarctica: King George Island [KU060818.1]
IW28	MG266399	Flavobacterium sp. NBRC 101333	98	stream water, Yakushima Island, Kagoshima, Japan[AB681458.1]	Uncultured bacterium clone W201e10_12765	98	Free Air CO2 Enrichment (FACE) field soil USA [JQ375009.2]
IW2	MG266400	Pseudomonas antarctica PAMC 27494	99	Freshwater Antarctica: King George Island[CP015600.1]	Pseudomonas antarctica strain PAMC 27494	99	Freshwater Antarctica: King George Island[CP015600.1]
IW4	MG266401	Pseudomonas antarctica PAMC 27494	99	Freshwater Antarctica: King George Island [CP015600.1]	Pseudomonas antarctica strain PAMC 27494	99	Freshwater Antarctica: King George Island [CP015600.1]
IW6	MG266402	Pseudomonas antarctica PAMC 27494	99	Freshwater Antarctica King George Island[CP015600.1]	Pseudomonas antarctica strain PAMC 27494	99	Freshwater Antarctica: King George Island[CP015600.1]
IW7	MG266403	Pseudomonas antarctica PAMC 27494	99	Freshwater Antarctica: Barton Peninsula, King George Island[CP015600.1]	Pseudomonas antarctica strain PAMC 27494	99	Freshwater Antarctica: Barton Peninsula, King George Island[CP015600.1]
IW8	MG266404	Pseudomonas yamanorum, TSA20	99	deep sea sediment Southern Indian Ocean[LT673850.1]	Pseudomonas yamanorum, strain TSA20	99	deep sea sediment Southern Indian Ocean[LT673850.1]
IW9	MG266405	Flavobacterium sp. HP11M	99	Amphibian skin, host:Pseudacris crucifer, USA [KM187393.1]	Uncultured bacterium clone	99	Human skin [HM263969.1]
IW10	MG266406	Janthinobacterium lividum TJ-1-35	99	Garden in Hamburg, Germany, HH100–HH107) [MF157598.1]	Uncultured bacterium clone	99	pit from Kuytun 51 Glacier, Tianshan Mountains, China [EU267871.1]
IW12	MG266407	Pseudomonas antarctica PAMC 27494	99	Freshwater Antarctica: Barton Peninsula, King George Island[CP015600.1]	Pseudomonas antarctica strain PAMC 27494	99	Freshwater Antarctica: Barton Peninsula, King George Island[CP015600.1]
IW13	MG266408	Pedobacter cryoconitis A37	99	bacteria in a wall crust in lava tube cave Iceland: [KF577526.1]	Pedobacter cryoconitis strain A37	99	bacteria in a wall crust in lava tube cave Iceland: [KF577526.1]
IW14	MG266409	Plantibacter sp. AK20-4	99	bacterial communities in alpine forest soils Austria [KP899147.1]	Plantibacter sp. AK20-4	99	bacterial communities in alpine forest soils Austria [KP899147.1]
IW15	MG266410	Flavobacterium collinsii 983-08	99	Farmed Fish (Oncorhynchus mykiss) liver [NR_145952.1]	Flavobacterium collinsii strain 983-08	99	Farmed Fish (Oncorhynchus mykiss) liver [NR_145952.1]
IW16	MG266411	Duganella sp. JH16	99	agricultural field frozen soil in winter, South Korea [KF424273.1]	Uncultured bacterium isolate 1112842459844	99	loamy sand of Eucalyptus forest in La Jolla, CA [HQ119381.1]
IW17	MG266412	Pseudomonas antarctica strain PAMC 27494	99	Freshwater Antarctica: King George Island [CP015600.1]	Pseudomonas antarctica strain PAMC 27494	99	Freshwater Antarctica: King George Island [CP015600.1]
IW18	MG266413	Variovorax paradoxus strain SL37	99	Endophyte in the circumpolargrass, Finland[KJ529023.1]	Variovorax paradoxus strain SL37	99	Endophyte in the circumpolargrass, Finland[KJ529023.1]
IW20	MG266414	Frondihabitans sp. BAR42	99	Water/rock samples Dry Valleys, Antarctica [KP717942.1]	Frondihabitans sp. BAR42	99	Water/rock samples Dry Valleys, Antarctica [KP717942.1]
IW21	MG266415	Pseudomonas fluorescens strain S18	99	Atlantic Salmon Eggs [KT223386.1]	Pseudomonas fluorescens strain S18	99	Atlantic Salmon Eggs [KT223386.1]
IW22	MG266416	Pseudomonas antarctica strain PAMC 27494	99	Freshwater Antarctica: King George Island [CP015600.1]	Pseudomonas antarctica strain PAMC 27494	99	Freshwater Antarctica: King George Island [CP015600.1]
IW23	MG266417	Pedobacter sp. R20-57	99	Alpine forest soil, Austria [KP899225.1]	Pedobacter sp. R20-57	99	Alpine forest soil, Austria [KP899225.1]
IW24	MG266418	Duganella sp. JH16	99	agricultural field frozen soil in winter, South Korea [KF424273.1]	Uncultured bacterium isolate	99	loamy sand of Eucalyptus forest in La Jolla, CA [HQ119381.1]
IW25	MG266419	Oxalobacteraceae bacterium PDD-69b-39	99	cloud water collected at the puy de Dome, 1465 m, France [KR922302.1]	Uncultured bacterium clone bar-b48	99	temperate highland grassland [JN024091.1]
IW27	MG266420	Pseudomonas fluorescens strain S18	99	Atlantic Salmon Eggs [KT223386.1]	Pseudomonas fluorescens strain S18	99	Atlantic Salmon Eggs [KT223386.1]
IW29	MG266421	Sphingomonas sp. strain ANT_H46B	99	soil sample from Arctowski Polish Antarctic Station [KY405918.1]	Uncultured Clostridium sp. clone ABLBf53	99	atmospheric boundary layer at 1500 feet [JF269141.1]
IW30	MG266422	Pseudomonas fluorescens strain S18	99	Atlantic Salmon Eggs [KT223386.1]	Pseudomonas fluorescens strain S18	99	Atlantic Salmon Eggs [KT223386.1]
IW31	MG266423	Pseudomonas yamanorum, strain TSA20	99	deep sea sediment Southern Indian Ocean [LT673850.1]	Pseudomonas yamanorum, strain TSA20	99	deep sea sediment Southern Indian Ocean [LT673850.1]
IW32	MG266424	Pseudomonas yamanorum, strain TSA20	99	deep sea sediment Southern Indian Ocean [LT673850.1]	Pseudomonas yamanorum, strain TSA20	99	deep sea sediment Southern Indian Ocean [LT673850.1]
IW33	MG266425	Sphingomonas aurantiaca strain MA101b	99	air- and dustborne Antarctic [NR_042128.1]	Sphingomonas aurantiaca strain MA101b	99	air- and dustborne Antarctic [NR_042128.1]
IW34	MG266426	Pseudomonas sp. BT-A-S4	99	isolated from Arctic, China (unpublished no further info) [KU671225.1]	Pseudomonas sp. BT-A-S4	99	isolated from Arctic, China (unpublished no further info) [KU671225.1]
IW35	MG266427	Pseudomonas fluorescens strain S18	99	Atlantic Salmon Eggs [KT223386.1]	Pseudomonas fluorescens strain S18	99	Atlantic Salmon Eggs [KT223386.1]
IW36	MG266428	Janthinobacterium sp. 1_2014MBL_MicDiv	99	Soil, Woods Hole, MA [CP011319.1]	Janthinobacterium sp. 1_2014MBL_MicDiv	99	Soil, Woods Hole, MA [CP011319.1]
IW38	MG266429	Pseudomonas frederiksbergensis strain AS1	99	arsenic-contaminated soil, South Korea: Gwangju, Kyunggi-Do [CP018319.1]	Pseudomonas frederiksbergensis strain AS1	99	arsenic-contaminated soil, South Korea: Gwangju, Kyunggi-Do [CP018319.1]

## Discussion

Our results demonstrate biomarker detection (nucleic acids) in environmental samples, as well as in conjunction with active microbial life detection methods through cultivation of microorganisms and a colourimetric microbial activity assay. The MinION generated metagenomic and genomic sequences identifying organisms across the 3 domains (Bacteria, Archaea, Eukaryotes) from environmental, enriched and an active-layer soil isolate (most closely related to *Pedobacter cryoconitis)* in a platform that was highly portable and robust in the highly remote and relatively extreme conditions in the Canadian high Arctic. The MinION sequencer is driven by Nanopore technology for real-time biological analysis where biomolecules are drawn through nanopore channels blocking and reducing the ionic current through the pore. The ionic current drop and transport duration provides discrimination between different nucleic acids, including base modifications such as methylation (Simpson et al., 2017). Nanopore technology offers considerable promise for low-resource overhead, miniaturized but robust astrobiology instruments. Because of these traits, it was used as the first DNA sequencer in space, on the ISS in 2016 (Castro-Wallace et al., 2016). In this study, we used the MinION to identify microorganisms across 3 domains based on nucleic acid sequences (Figure 2) in the Canadian high Arctic. We have not attempted to quantify error rates in this study, though high error rates in basecalling remains a serious concern with the Nanopore MinION (Deschamps et al., 2016) with current base calling error rates of 13–15% (Boža et al., 2017). Nevertheless, we found the data from MinION to be comparable with Illumina sequencing in environmental and enriched samples (Figure 3) with similar proportions of phyla detected using both technologies. Similarly, Brown et al. (2017) were able to correctly assign MinION generated reads from synthetic metagenomes (a mixture of genomic DNA from 20 bacterial strains) to the correct bacterial species up to 98% of the time using the high quality 2D reads (Brown et al., 2017).

Overall, both advances in the nanopore sequencing hardware technology and better bioinformatics tools for MinION-generated sequences should continue to improve sequencing accuracy in the coming years (Boža et al., 2017; Brown et al., 2017). For example, the Oxford Nanopore is currently developing and testing the SmidgION, using MinION sequencing technology and approximately the size of a standard USB memory stick and requiring less energy. While astrobiology life detection *per se* may not need highly accurate reads as opposed to the ability to recognize biogenically produced patterns, a certain level of sequence accuracy will be required in order to discern between earth microbial contaminants from extant alien organisms which share an origin with or from organisms on Earth. In addition, the detection of an unambiguous complex molecular biomarker such as DNA or RNA in samples from Mars, Europa and/or Enceldadus would in itself be a revolutionary scientific discovery if the platform can be designed with the proper controls (i.e., with blank samples) and protocols to detect terrestrial contamination.

The relatively very low weight and low energy requirements of the Nanopore MinION platform for in situ nucleic acid sequencing is beneficial for life detection missions elsewhere, but also to address planetary protection concerns—both for “forward” contamination in future robotic missions within our solar system, and to assess the potential for “backward” contamination prior to sample return to Earth (Race et al., 2015). The lower limits of biomass detection in environmental samples remains to be tested with the MinION. The current 2017 Nanopore MinION low input kit (SQK-RLB001) requires a minimum of 1 ng of DNA for successful sequencing which would require ~2.5–5 × 10^5^ cells per gram or ml environmental sample based on a 4–2 Mbp microbial genome mass of ~2.5–5 fg per genome. In most cases this limit will be determined by the efficiency of nucleic acid extraction protocols which is often sample specific. Nonetheless, robust DNA extraction and nucleic acid sequencing has been successfully carried out on a number of low biomass/analog samples on Earth (Navarro-González et al., 2003; Direito et al., 2012; Goordial et al., 2016a; Mojarro et al., 2017) and can be used to inform biomarker extraction elsewhere. Ideally however, nanopore or other novel future novel sequencing technologies that can successfully sequence even lower amounts of DNA [0.1 (= ~10^4^ cells/g or ml) to 0.01 ng (= ~10^3^ cells/g or ml)] from extremely low biomass samples would be more desired for astrobiology life detection instrument platforms given the expected very low concentrations that would probably exist in Mars, Europa or Enceladus samples. Current and future such technologies should be field and lab tested in extremely low biomass terrestrial analog samples such as Antarctic high elevation dry valley permafrost soils (Weber and Legge, 2009) or Atacama extremely dry desert soils (Goordial et al., 2017). In principle, the MinION methodology as outlined here could be automated, which has not been explored here. SetG is an example system which uses the MinION in conjunction with a custom Claremont BioSolutions SimplePrep X1 Automated Lysis and Nucleic Acid Extraction Platform (Mojarro et al, 2016). There are several promising technologies and instruments for such an application including the Voltrax from Oxford Nanopore, or the SOLID-SPU associated with the SOLID life detection instrument (Parro et al., 2011; Manchado et al., 2015).

In this study, we also showed how wells from a Biolog plate resulting in growth can be enriched for extant microbial communities and subsequently have nucleic acids extracted and the communities sequenced—which offers many possibilities for substrate utilization experiments for life detection and ecological studies. The redox dye chemistry employed by the Biolog Ecoplate can be made high throughput, miniaturized, and a number of substrates can be simultaneously tested including chiral forms of sugars and amino acids. Additionally, tolerance and resistance to a number of different environmental conditions such as pH, salinity, metals, antibiotics etc. can be tested simultaneously to probe the CLPP of any extant life encountered amenable to enrichment in this manner. Since positive wells are indicative of metabolism and possibly growth, each positive well represents an enrichment of active microorganisms that can then be used for further analysis—for sequencing, or possibly isolation. The NASA Biosentinel mission, projected to launch in November 2018 as a secondary payload mission on the new Space Launch System, will have a similar microfluidic microbial activity assay (using LED detection system and the metabolic indicator dye alamarBlue, similar to Biolog chemistry) to test the effects of radiation on a targeted yeast strain viability in (see https://www.nasa.gov/centers/ames/engineering/projects/biosentinel.html).

While molecular techniques (ex. metagenomic sequencing) provide some information independent of our ability to culture microorganisms, it is essentially impossible to confirm new gene and pathway functions from pure sequence data. A true understanding of the adaptations, physiology, and metabolic capacities of the bacteria inhabiting the extreme cryoenvironment analog sites requires their cultivation in the laboratory. Recently developed procedures, such as the iChip method can grow up to 20–30% of the microbes, many of which have been found to be novel (Nichols et al., 2010). One such recently discovered organism first cultured with the ichip, *Eleftheria terrae* is the first in 25 years capable of producing a new class of antibiotic (teixobactin) (Ling et al., 2015). The cryo-iplate is modeled off of the ichip method, utilizing diffusion of *in situ* nutrients across a 0.03 membrane into a solid media (gellan gum) to form a medium that more closely mimics in situ conditions. Using this methodology, the majority of the isolates from the ice wedge soil were related to organisms previously isolated (Table 2); though few candidates for novel strains and species were identified, one of which we sequenced. While there is utility in isolating microorganisms, and a culturable isolate would be an unambiguous sign of life if proven not to be a contaminant, this methodology would be very difficult to robotocize, and may be limited to future human missions, for example to Mars. The small volume involved for each of these modules (Biolog plate, MinION, cryo-iplate) used in this study means that a single sample (for example a core segment or scoop full of soil) can be split for multiple analyses occurring in parallel.

The methods in this study were used in a life detection/astrobiology context, however all methodology presented here has clear application to microbial ecology work, especially *in situ*. The single molecule long reads generated by the MinION proved to be a valuable tool to probe the microbial ecology of analog permafrost samples at the single organism level, offering the ability able to place individual functional genes in genomic context. The long reads from the metagenome allows single functional genes to be placed in genomic context within the organism it was sequenced from, without any potential bias from assembling methodology (Alkan et al., 2011; English et al., 2012; Koren and Phillippy, 2015). Long read sequencing technologies (ex- Moleculo, PacBIO) have been recently applied to generate soil (White et al., 2016) and skin (Tsai et al., 2016) metagenomes, and have proven advantageous to address the challenges of assembly and genomic binning in samples with low coverage regions or with diverse microbial communities. Similarly, other long read generating technology such as PacBIO have been found to be especially advantageous resolving genomic context when multiple replicons and large repetitive elements are present that make it inherently difficult to assemble by short read sequencing technologies (Ricker et al., 2016). Though not the focus of this paper, we were able to identify such a repetitive sequence (15 bp long) associated with an integron integrase in a 2,850 bp single molecule read from the MinION-generated ice wedge soil metagenome. This technology also allows comparison of long stretches of DNA from environmental metagenomic samples to known isolate genomic DNA, such as the homology found in this study between an enriched *Pseudomonas*, and *P. Antarctica* PAMC27294 (Figure S1). This enables science such as genomic comparisons with related isolates in the database to examine questions sensitive to single amino acid differences such as looking at cold adaptation traits which aid activity in the permafrost environment (Goordial et al., 2016b) providing an invaluable window into single organism genomes when single cell methodologies are not feasible, or when isolating microorganisms is not possible. Real-time DNA sequencing has been carried out in the field previously in polar environments (Edwards et al., 2016; Johnson et al., 2017); for example, in the Antarctic Dry Valleys where hand warmers and insulating materials were used to run the device for 2 h outside of shelter (Johnson et al., 2017). The use of fast, simple and reliable tools for detection and identification of microbial life will help researchers respond to rapidly changing conditions and communities, or help direct hypothesis while in the field instead of post analysis in the lab. This is especially important in remote and hard to sample areas.

## Conclusions

Our results demonstrated successful biosignature detection (DNA) using nanopore sequencing on Mars analog environmental samples, in conjunction with active life detection methods through cultivation of microorganisms and a colorimetric microbial activity assay in a highly remote and extreme terrestrial environment. The platforms and methods outlined here are highly portable and offer the possibility to examine microbial ecology in real-time, in dynamic, and remote environments. Future planetary exploration life detection missions will likely not rely on a single instrument, rather a suite of instruments will need to be used in concert to confirm whether extant or relic life is present. The tools presented here are pre-existing, relatively low weight, have relatively low energy requirements, and relatively low cost. While Nanopore MinION sequencing technology has considerable promise and potential as an unambiguous biosignature detection platform for future planetary exploration missions, a number of significant hurdles remain. To optimize these tools for a flight mission further work needs to be done; for example, the lifetime of protein based nanopores are currently not suitable for the long duration space flights necessary for targets like Mars, Europa or Enceladus; however, synthetic solid state nanopore sequencers are currently being developed through the NASA ColdTech Program (Whyte personal communication). It will also be crucial to develop miniaturized robust, robotic nucleic acid extraction platforms capable of extracting sufficient nucleic acids from a variety of very low biomass Mars and Icy moon analog samples for successful nanopore sequencing technologies, current and future.

## Author contributions

JG carried out field work, lab work, analysis, and writing, IA carried out field work and writing, KC-Y carried out Illumina Sequencing, EM and KH carried out isolation and sanger sequencing and analysis, and LW advised and contributed significantly to writing and analysis.

### Conflict of interest statement

JG received a $1000 travel bursary from Oxford Nanopore to aid attendance costs at the Astrobiology Science conference (AbSciCon) in Mesa, Arizona, April 23–27th, 2017, where she gave an oral presentation on these results. ONT have generously provided free-of-charge reagents and technical support for this project. No authors were/are financially compensated by ONT, hold stocks, shares or options. The other authors declare that the research was conducted in the absence of any commercial or financial relationships that could be construed as a potential conflict of interest.
