# Supplementary figures and images for: In Situ Field Sequencing and Life Detection in Remote (79°26′N) Canadian High Arctic Permafrost Ice Wedge Microbial Communities
Source: Front Microbiol. 2017 Dec 20;8:2594. doi: 10.3389/fmicb.2017.02594 (PMC5742409; doi:10.3389/fmicb.2017.02594)

Figure S1. Citric Acid Cycle genes found in MinION generated metagenome

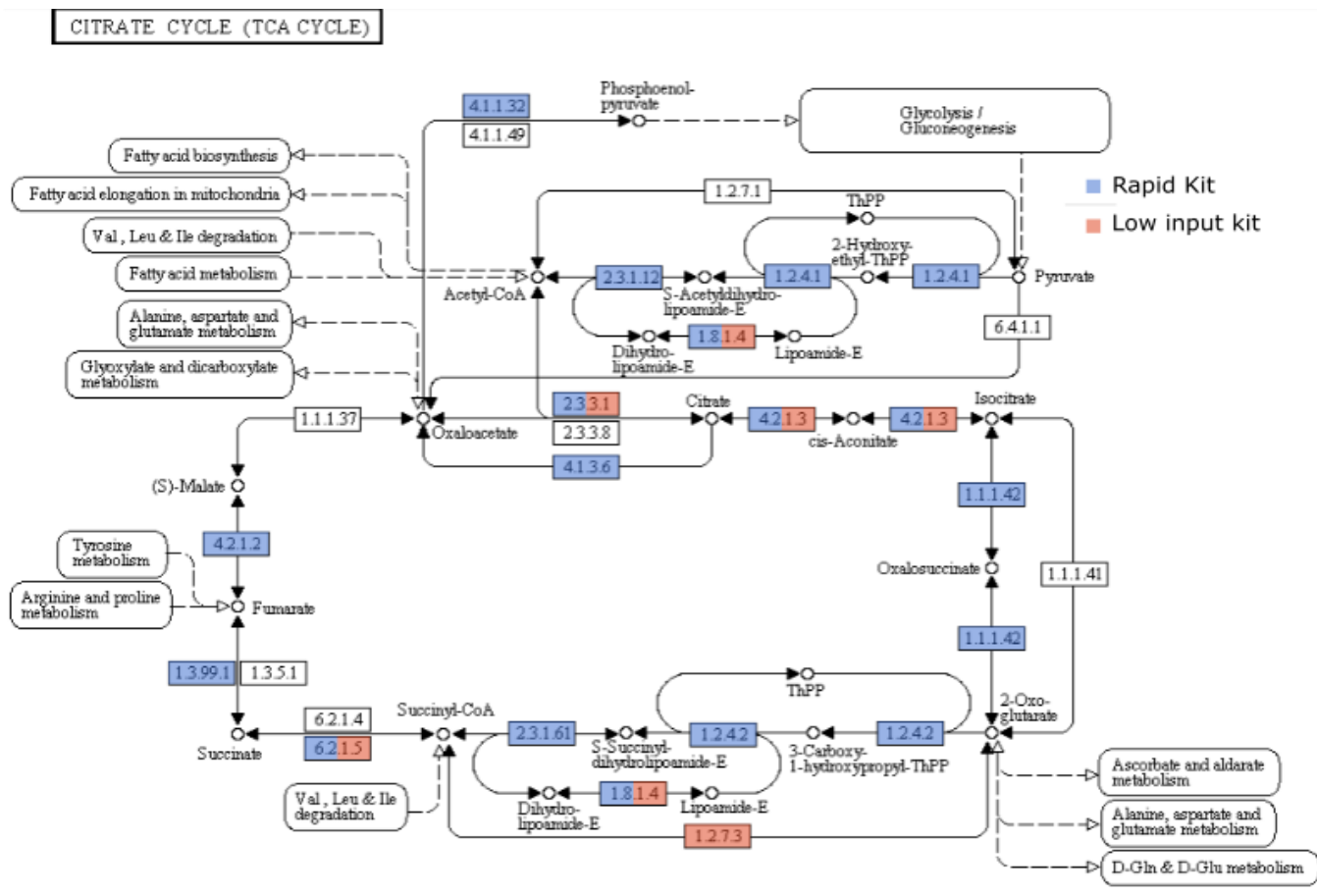

Supplement: Supplementary file 1 [file Image1.PDF]

**Figure S3. *Pedobacter* sp. IW39 read length distribution**

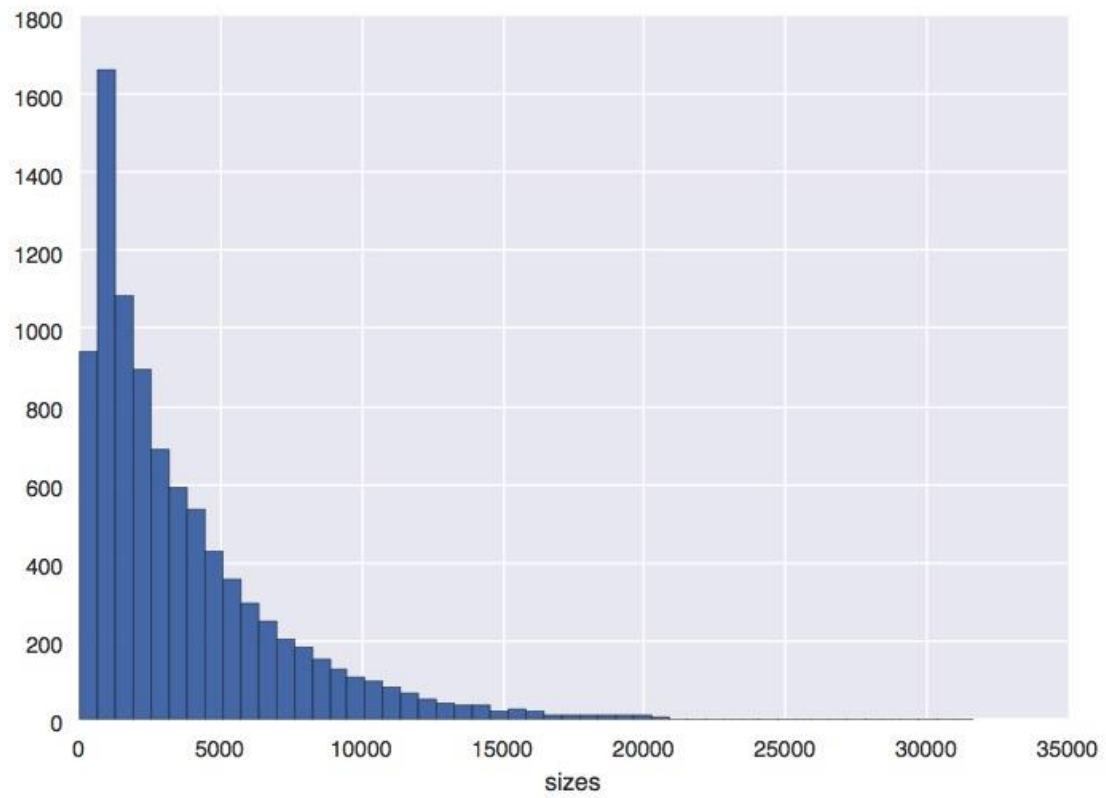

Supplement: Supplementary file 3 [file Image3.pdf]
