# Supplementary material for: In Situ Field Sequencing and Life Detection in Remote (79°26′N) Canadian High Arctic Permafrost Ice Wedge Microbial Communities
Source: Front Microbiol. 2017 Dec 20;8:2594. doi: 10.3389/fmicb.2017.02594 (PMC5742409; doi:10.3389/fmicb.2017.02594)

Figure S2. Serine transporter and adjacent homology of single molecule long read to *P. Antarctica* PAMC27294

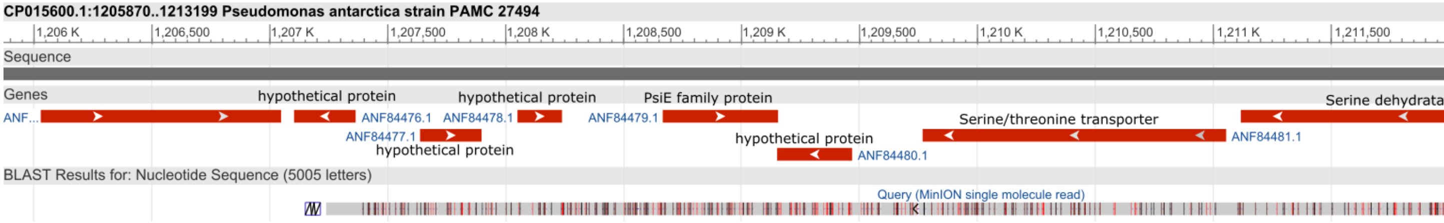

Supplement: Supplementary file 2 [file Image2.PDF]
