# Supplementary material for: In Situ Field Sequencing and Life Detection in Remote (79°26′N) Canadian High Arctic Permafrost Ice Wedge Microbial Communities
Source: Front Microbiol. 2017 Dec 20;8:2594. doi: 10.3389/fmicb.2017.02594 (PMC5742409; doi:10.3389/fmicb.2017.02594)
Supplement: Supplementary file 4 [file Table1.DOCX]

**Table S1. MinION metagenomic sequencing statistics**

| **Sample** | **Kit Used** | **No. of Seqs after QC** | **Mean length (bp)** | **Mean GC %** | **No. of ribosomal features (predicted/ known)** | **No. of predicted proteins features (predicted/ identified)** | **MG-RAST ascension number** |
| --- | --- | --- | --- | --- | --- | --- | --- |
| **Ice wedge soil (12 hour run)**  **Ice Wedge soil** | Low input kit (only 2D reads) | 2,116 | 3,811 ± 2,704 | 51 ± 7 % | 117/32 | 8,823/1,523 | mgm4705800.3 |
|  | Low input kit (All reads) | 6,348 | 3,843 ± 2,732 | 51 ± 5 % | 244/37 | 29,318/1,539 | mgm4705797.3 |
| **Ice wedge soil (48 hour run)** | Rapid kit | 9,530 | 3,018 ± 2,015 | 58 ± 11% | 14/11 | 30,629/8,150 | mgm4735353.3 |
| **Biolog L-serine Wells (48 hour run)** | Rapid kit | 2,085 | 2,267 ± 2,496 | 62 ± 7 % | 10/9 | 4,694/1,481 | mgm4718581.3 |
