# Supplementary material for: In Situ Field Sequencing and Life Detection in Remote (79°26′N) Canadian High Arctic Permafrost Ice Wedge Microbial Communities
Source: Front Microbiol. 2017 Dec 20;8:2594. doi: 10.3389/fmicb.2017.02594 (PMC5742409; doi:10.3389/fmicb.2017.02594)
Supplement: Supplementary file 5 [file Table2.DOCX]

Table S2. Cold Adaptation Proteins in MinION generated metagenomes

| **function** | **1Drapidkit_pass** | **2D_pass_wedgelowinputkit** | **wedgebiolog_rapidkit** |
| --- | --- | --- | --- |
| Cold shock protein CspA | 0 | 3 | 0 |
| Universal stress protein F | 1 | 0 | 0 |
| Glycine betaine/L-proline ABC transporter, glycine betaine/L-proline- binding/permease protein | 0 | 0 | 1 |
| High-affinity choline uptake protein BetT | 0 | 0 | 3 |
| Sarcosine oxidase beta subunit (EC 1.5.3.1) | 0 | 0 | 1 |
| L-proline glycine betaine ABC transport system permease protein ProV (TC 3.A.1.12.1) | 0 | 0 | 1 |
| L-proline glycine betaine binding ABC transporter protein ProX (TC 3.A.1.12.1) | 1 | 0 | 0 |
| Choline dehydrogenase (EC 1.1.99.1) | 1 | 0 | 0 |
| Peroxidase (EC 1.11.1.7) | 1 | 1 | 0 |
| Catalase (EC 1.11.1.6) | 3 | 1 | 5 |
